# Water and beverage consumption patterns among 4 to 13-year-old children in the United Kingdom

**DOI:** 10.1186/s12889-017-4400-y

**Published:** 2017-05-19

**Authors:** Florent Vieux, Matthieu Maillot, Florence Constant, Adam Drewnowski

**Affiliations:** 1 MS-Nutrition, Faculté de Médecine La Timone, Marseille, France; 2 Nestlé Waters M.T, 12 boulevard Garibaldi, 92130 Issy-les-Moulineaux, France; 3 Center for Public Health Nutrition, University of Washington, 305 Raitt Hall, 4000 15th Ave NE, Box 353410, Seattle, WA 98195 USA

**Keywords:** Water, tap and bottled, Beverages, Consumption patterns, Children, Adequate water intake, EFSA recommendation

## Abstract

**Background:**

The UK government has announced a tax on sugar-sweetened beverages. The aim of this study was to assess consumption patterns for plain drinking water relative to sugary beverages among UK children.

**Methods:**

Dietary intake data for 845 children aged 4–13 years came from the nationally representative cross-sectional National Diet and Nutrition Survey, 2008–2011. Beverage categories were drinking water (tap or bottled), milk, 100% fruit juices, soda, fruit drinks, tea, coffee, sports drinks, flavored waters, and liquid supplements. Consumption patterns were examined by age group, gender, household incomes, time and location of consumption, region and seasonality. Total water consumption from drinking water, beverages, and foods, and the water-to-calorie ratios (L/kcal) were compared to the EFSA (European Food Safety Authority) adequate intake standards.

**Results:**

Total water intake (1338 ml/d) came from plain water (19%), beverages (48%), and food moisture (33%). Plain drinking water provided 258 g/d (241 g/d for children aged 4–8 years; 274 g/d for 9–13 years), mostly (83.8%) from tap. Water and beverages supplied 901 g /d of water. Tap water consumption increased with income and was highest in the South of England. The consumption of bottled water, soda, tea and coffee increased with age, whereas milk consumption declined. About 88.7% of children did not meet EFSA adequate intake standards. The daily water shortfall ranged from 322 ml/d to 659 ml/d. Water-to-calorie ratio was 0.845 L/1000 kcal short of desirable levels of 1.0–1.5 L/1000 kcal.

**Conclusion:**

Total water intake were at 74.8% of EFSA reference values. Drinking water consumption among children in the UK was well below US and French estimates.

## Background

Water requirements to meet hydration needs are usually supplied by plain drinking water, water from beverages, and by moisture from food [1–3]. Water and beverages generally supply 65–75% of total water, whereas food moisture supplies 25–35%, depending on eating habits and age [4, 5]. Water consumption patterns can be further influenced by cultural habits and by access to drinking water [1].

The European Food Safety Authority (EFSA), has issued Dietary Reference Values (DRV) for water that were based, in part, on observed population intake of plain drinking water (tap and bottled); water from caloric and non-caloric beverages, and from food moisture. The EFSA adequate intake were set at 1600 ml/d for boys and girls aged 4–8 years; at 1900 ml/d for girls and at 2100 ml/d for boys aged 9–13 years [1]. The EFSA DRVs have been used as goals for individual intake [1]. Based on water-to-energy ratios, the desirable total water intake in children should be in the 1.0 to 1.5 L/1000 kcal range [1].

There is a growing literature on the consumption of plain drinking water by children and youth in the US [5–7], Mexico [8, 9], Germany [10, 11], Belgium [11, 12], and France [11, 13] and more recently in other countries such as Brazil, Argentina, Uruguay, Spain, Poland, UK, Turkey, Iran, China and Indonesia [8]. That literature attests to the importance of replacing caloric beverages in the diets of children with plain drinking water [5, 14, 15]. Given the high consumption of added sugars in the UK, the study of water consumption patterns (from plain water, beverages and foods) among UK children assumes a particular relevance.

The present goal was to examine the consumption of plain drinking water by children and youth in the UK, as distinct from the consumption of caloric beverages [15–17]. In this study, the nationally representative NDNS database (National Diet and Nutrition Survey 2008–2011) was used to assess total water consumption among British children aged 4–13 years. The NDNS is the principal national dietary survey for the UK that is jointly funded by Public Health England (PHE), an executive agency of the Department of Health, and by the UK Food Standards Agency (FSA). The NDNS is carried out by a consortium of three organizations: NatCen Social Research (NatCen), MRC Human Nutrition Research (HNR) and the University College London Medical School (UCL) [18].

Analyses reported below examined water consumption patterns by gender and age, socio-demographics characteristics, and by time and place of consumption. Additional analyses estimated total water sources (plain water, beverages, and foods) and the water-to-calorie ratios (L/kcal) in relation to the EFSA desirable norms. The NDNS is highly relevant to policy making; the data are used by the UK Government to develop policy and monitor progress on diet and nutrition objectives. This study was intended to inform one of those objectives related to the provision of safe, clean drinking water in schools [19].

## Methods

### Dietary intake database: The National Diet and Nutrition survey (NDNS)

The NDNS uses 4-day food records to provide estimates of diet, nutrient intake, and nutritional status for a representative sample of the UK population aged 1.5 years and older. The present pooled database was for three cycles of cross-sectional data collection (2008/09, 2009/10, 2010/11). For each year, participants were asked to record food and drinks consumed both at home and away from home in diaries during 4 days, including weekend days. Data collection methods remain constant from year to year allowing data to be combined across survey years. Children aged 12 years and over were encouraged to complete the diaries themselves, while for children below 12 years the parent or caregiver was asked to complete the diary. The survey also included an interview to provide information on socio-demographic status and lifestyle [18].

Heights and weights were measured, using a portable stadiometer and weighing scales, the details of which are provided elsewhere [18]. Parents/guardians provided the written consent for taking measurements. Body Mass Index (BMI) values (kg/m^2^) were calculated and compared to pediatric standards [20]. The NDNS data were collected for a total sample of 3073 individuals, including 845 children aged 4–13 years. Ethical approval for NDNS was obtained from the Oxfordshire Research Ethics Committee**.** The consent form is available online [21].

### Age, gender, and sociodemographic status

The two age groups were 4–8 years and 9–13 years old, corresponding to previous studies [5]. Regions of residence were defined as North, Central/Midlands, South (incl. London), and Scotland, Wales, and Northern Ireland. Ethnic groups were separated into white and non-white.

Household incomes were self-reported. Reported household incomes were first divided by an adjusted number of persons in the household to arrive at income-per-person. The income per consumption unit (IUC) was estimated by divided the total household income by the number of consumption unit of the household. The scale used to define a consumption unit was defined by the Organisation for Economic Co-operation and Development (OECD) [22] and is the most widely used at present [23]. It is measured as follows: one unit for the first adult, 0.5 for the others individuals of more than 14 years old and 0.3 for individuals of less than 14 years old. Then, the IUC was divided by quartiles.

### Plain water and beverage consumption

Water and beverages were classified into these categories: tap water, bottled water, milks (including flavored), sodas (regular and diet), 100% fruit juices, hot beverages (coffee and tea), fruit drinks, sports and energy drinks, flavored waters, and liquid supplements for nutrition use. All other foods were categorized as solid food.

Following past procedures [5], a distinction was made between the consumption of drinking water and beverages (in g/d) and the consumption of water from water, beverages, and foods (in ml/d). The NDNS food records for each respondent provided information on the amount in grams of each food and beverage consumed. The water content of beverages and the moisture content of foods were then established using a nutrient composition database [24].

Analyses of water and beverage consumption by eating occasion and eating location were based on the mean total weight of plain water and water from beverages calculated in g/d. By contrast, the comparisons to EFSA total water guidelines were for ml of water content from plain water, beverages, and foods, calculated in ml/d.

Data on the time of consumption were also provided in the NDNS. The time of consumption was assigned to one of 7 categories: 6 am–9 am, 9 am-12 pm, 12 pm- 2 pm, 2 pm–5 pm, 5 pm–8 pm, 8 pm–10 pm and 10 pm-6 am. Time analyses were based on the entire sample; those children who consumed nothing during a given time period were assigned 0 g consumption.

Information on the place of food or beverage consumption was also examined. The key places reported were “at home”, “at school”, “friends”, “fast-food”, “coffee and restaurant”, “other”, and “unspecified”. Analyses by place were conducted only for those children who declared that location as place of consumption. There could be multiple locations per child.

### Statistical analyses

Analyses evaluated the survey-weighted mean 4-day water intake overall and by age-group, gender, and IUC. The consumption of plain water, tap and bottled, was evaluated separately for the entire population and by age-group, gender and IUC. The consumption of water and beverages (milk, sodas, juice, hot drinks, fruit drinks, sport and energy drinks, flavored water and supplements), and total water intake from beverages and foods were estimated by socio-demographic status, seasonality, BMI, ethnicity, region, the time and the place of consumption. Total water intake from water, beverages, and solid foods were estimated in ml/d for the total population and subgroups of interest.

All analyses by gender, age, IUC, season, BMI, ethnic group and region were based on ANOVAs. Post-hoc comparisons between means using Bonferroni correction were performed where indicated. Tests of percentages of children failing to meet EFSA recommendations were based on non-parametric Chi square test. The estimated percent of children meeting the Dietary Reference Intake (DRI) for water represents the upper bound of the estimate, since the mean of 4 days’ water intake may not represent the habitual intake of an individual. Total water intake per 1000 kcal and total energy intake from food groups were compared by gender and age. An additional multiple regression was conducted to examine the effect of IUC on water consumption, adjusting for covariates. The tests were conducted using the SAS statistical software package version 9·4 and the SURVEYREG, SURVEYMEANS and SURVEYFREQ procedures.

Total water intake were expressed as percent EFSA guideline (1600 ml/d for boys and girls 4–8 years; 2100 ml/d for boys 9–13 years and 1900 ml/d for girls 9–13 years). The percent of children not meeting EFSA guidelines was calculated. Associations between age, gender and compliance with EFSA standards were tested with a non-parametric Chi square test. Statistical significance level was 0.01 to take multiple statistical tests into account [25].

## Results

### Plain water consumption

The consumption of plain water (ml) by age, gender, and socio-demographic group is shown in Table 1. On average, children aged 4–13 years drank a total of 258 ml/d of water. Most of the water (83.8%) came from the tap. Overall, children consumed 216 ml tap water and only 42 ml bottled water. Older children drank more bottled water than did younger children (54 ml vs 29 ml). There was no significant difference between boys (234 ml) and girls (283 ml) in terms of water intake.

**Table 1:** The consumption of tap and bottled water (ml/d) by children in the NDNS sample (*n* = 845) by age group, gender, and socio-demographic group. The data are means and standard deviations (Std)

	Water (Tap + Bottled)			Tap water		Bottled water	
	*N*	Mean	Std^1^	Mean	Std^1^	Mean	Std^1^
All	845	257.6	275.7	215.9	259.8	41.7	116.2
Age (years)							
4–8	436	241.0	245.4	212.4	228.0	28.6	94.9
9–13	409	273.7	304.0	219.3	290.1	54.4	134.0
		0.1061		0.7018		**0.0041**	
Gender							
Boys	430	233.7	247.6	192.8	229.5	40.9	118.0
Girls	415	282.7	300.3	240.1	286.0	42.5	114.4
		0.0284		0.0275		0.8543	
Quartiles of IUC^2^							
Q1 (poorer)	148	177.1	193	145.8	175.9	31.3	97.0
Q2	186	260.7	282.6	208.7	249.9	52.0	135.6
Q3	215	272.6	256.4	240.0	251.5	32.6	96.6
Q4 (richer)	197	318.9	331.8	266.5	319.4	52.4	125.6
		**<0.0001** ^**a**^		**<0.0001** ^**a**^		0.2319	
Season							
Winter	230	241.8	224.8	208.8	212.7	33.0	92.6
Spring	233	286.7	308.2	234.0	280.0	52.8	138.8
Summer	207	242.0	238.2	194.7	225.1	47.3	119.1
Fall	175	254.3	326.3	223.9	319.5	30.4	105.9
		0.3518		0.4019		0.119	
BMI							
Under/normal	607	261.0	282.4	221.2	268.9	39.8	112.3
Overweight/obese	204	251.8	250.9	208.6	236.1	43.2	116.5
		0.6973		0.5803		0.7080	
Ethnic group							
White	723	247.1	270.4	205.0	254.7	42.1	118.1
Non white	122	310.7	299.7	271.1	281.6	39.6	104.5
		0.0276		0.0123		0.8156	
Region							
England: North	201	234.4	233.5	178.2	208.4	56.2	137.4
England: Central/Midlands	142	207.3	240.1	184.4	229.0	22.9	88.8
England: South (incl. London)	355	303.6	317.4	264.2	305.0	39.4	112.0
Scotland,Wales, Northern Ireland	147	210.9	232.2	165.8	206.9	45.1	115.9
		**0.0016** ^**bc**^		**0.0003** ^**bd**^		0.0872	

^a^Significant two by two differences: Q3 vs Q1; Q4 vs Q1

^b^Significant two by two differences: England South vs Scotland, Wales, Northern Ireland

^c^Significant two by two differences: England Central vs England South

^d^Significant two by two differences: England North vs England South

^1^Std^:^ Standard Deviation

^2^IUC: Income per Consumption Unit

Comparisons between two means were either significant (bold) or not

Table 1 also shows that the consumption of tap water (but not bottled water) rose sharply with income. Whereas children in the lowest IUC quartile consumed 146 ml/d, those in the highest quartile consumed 267 ml/d. Most tap water was consumed in the South of England, including London (264 ml/d), significantly more than in the North (178 ml/d) or in Scotland, Wales and Northern Ireland (166 ml/d). The effect of income quartiles on the consumption of total water (bottled and tap) and for tap water remained significant in multiple regression models, adjusted for age, gender, BMI classes, ethnicity and region (data not shown). In every case, the quartile Q1 was significantly different from quartiles Q3 and Q4.

There were no significant differences by gender, BMI status, or seasonality for tap or bottled water consumption. Non-white participants consumed more tap water (271 vs 205 ml) but not significantly (*p* = 0.012).

### Consumption of plain water and other beverages

Table 2 shows that the combined consumption of plain water and beverages was 953.2 g. Older children drank more than did younger children (1008 g vs 897 g). There was no difference in total beverages consumption between boys and girls. There was a significant effect of income and a weaker effect of overweight.

**Table 2:** The consumption of water and beverages by by children in the NDNS sample (*n* = 845) by age group, gender, and socio-demographic group. The data are means and standard deviations (Std)

		All beverages including water		Milk		Soda		Juice		Tea and coffee		Fruit drink		Sport and energy drink		Flavored water		**Supplement**	
	*N*	Mean	Std^1^	Mean	Std^1^	Mean	Std^1^	Mean	Std^1^	Mean	Std^1^	Mean	Std^1^	Mean	Std^1^	Mean	**Std** ^**1**^	**Mean**	**Std** ^**1**^
All	845	953.2	367.9	212.3	169	89.3	145.9	91.8	121.8	34.7	81.7	242.1	248.3	12.1	52.0	12.2	53.2	1.1	29
Age (years)																			
4–8	436	896.5	313.1	240.5	158.9	46.7	92.0	91.3	114.0	23.6	60.2	241.3	238.1	3.9	29.2	8.2	44.3	0.0	0.0
9–13	409	1008.3	410.9	184.9	174.7	130.8	176.8	92.3	129.6	45.4	98.6	242.8	258.9	20.1	67.4	16.2	61.0	2.1	41.7
		**<0.0001**		**<0.0001**		**<0.0001**		0.912		**0.0003**		0.9351		**<0.0001**		0.0389		0.1614	
Gender																			
Boys	430	969.2	362.3	229.2	179.9	92.0	162.2	99.2	133.0	35.7	88.4	249.9	241.7	14.0	52.8	14.4	58.4	1.21	31.6
Girls	415	936.3	373.3	194.6	155.2	86.5	127.0	84.1	108.5	33.6	74.4	233.8	255.0	10.1	51.1	10.0	47.1	1.0	26.2
		0.2265		**0.0058**		0.5819		0.0801		0.7393		0.3727		0.2988		0.2455		0.8717	
Quartiles of IUC																			
Q1 (poorer)	148	863.8	338.9	213.9	166.3	85.3	136.4	72.2	112.4	58.2	119.2	238.9	233.7	11.4	54.0	6.9	35.7	0.0	0.0
Q2	186	991.0	401.8	204.5	176.0	105.9	157.2	69.4	90.2	31.2	65.0	290.1	273.8	14.3	59.4	12.7	60.0	2.2	39.1
Q3	215	964.6	339.4	214.5	168.9	91.1	154.6	105.7	136.9	32.8	82.9	227.0	246.1	9.5	44.0	11.3	46.5	0.0	0.0
Q4 (richer)	197	1021.2	392.7	211.2	166.7	94.9	150.1	115.7	133.4	26.6	69.3	226.8	244.6	8.7	40.9	15.5	60.2	2.8	46.7
		**0.0005** ^**a**^		0.9570		0.6828		**0.0004** ^**b**^		0.0559		0.1317		0.7031		0.3994		0.3710	
Season																			
Winter	230	920.2	314.5	213.7	162.7	83.1	146.2	111.2	131.2	27.7	65.0	219.9	224.2	8.2	39.3	12.1	49.6	2.5	43.2
Spring	233	1000.1	385.3	212.8	169.1	101.1	149.4	91.8	120.7	31.1	85.5	247.9	240.9	14.4	62.3	14.2	61.6	0.0	0.0
Summer	207	977.4	376.5	214.8	170.5	93.1	144.0	72.0	109.5	44.4	82.6	276.7	290.5	19.1	65.2	15.4	63.2	0.0	0.0
Fall	175	900.3	390.3	207.2	176.7	76.2	142.7	92.0	121.3	36.3	94.0	220.3	230.0	5.5	27.2	6.2	24.8	2.2	40.3
		0.0283		0.983		0.2732		0.042		0.2071		0.1487		0.0556		0.0495		0.3657	
BMI																			
Under/normal	607	944.4	372.0	216.0	170.8	88.3	145.5	95.5	127.1	30.4	76.8	228.5	246.3	11.9	51.8	11.2	48.4	1.5	34.2
Over/obese	204	976.8	355.1	196.3	162.9	93.3	149.8	80.5	106.1	48.5	96.0	276.5	246.4	13.2	54.6	16.7	67.3	0.0	0.0
		**0.0012**		0.1811		0.7121		0.1081		0.0348		0.0431		0.7841		0.2814		0.1629	
Ethnic group																			
White	723	970.8	359.3	213.3	171.0	93.5	149.2	94.1	125.2	35.6	84.4	259.2	254.3	12.6	53.4	14.1	56.7	1.3	31.4
Non white	122	863.3	402.9	201.6	141.6	88.2	142.7	113.8	103.2	35.7	61.1	241.3	239.9	10.0	44.9	1.6	10.8	0.0	0.0
		0.0114		0.7432		0.0508		0.2160		0.4330		**<0.0001**		0.4367		**0.0004**		0.1625	
Region																			
England: North	201	934.3	329.8	227.5	179.0	70.7	100.8	99.0	134.1	27.5	65.9	249.8	232.1	12.6	56.0	12.8	50.1	0.0	0.0
Central/Midlands	142	966.3	378.4	202.2	157.2	90.7	143.5	98.3	125.0	59.1	124.1	289.1	261.8	11.2	45.4	8.4	44.5	0.0	0.0
England: South	355	938.8	379.8	192.3	155.8	87.8	150.1	90.1	117.0	29.3	70.6	217.7	253.7	9.4	44.0	7.5	46.2	1.0	28.3
Scotland,Wales, Northern Ireland	147	1008.3	375.7	226.1	151.5	110.2	153.4	78.5	103.3	26.1	63.8	243.9	244.7	9.2	36.1	33.0	74.2	0.0	0.0
		0.2661		**0.0014** ^**c**^		0.0142		0.5772		0.0252		0.0268		0.4411		0.0265		0.3698	

Data for beverages are by beverage type

^a^Significant two by two differences: Q1 vs Q4

^b^Significant two by two differences: Q2 vs Q4

^c^Significant two by two differences: South vs Scotland Wales and N Ireland

^1^Std^:^ Standard Deviation

Comparisons between two means were either significant (bold) or not

Different age, gender, ethnicity, and socioeconomic effects were obtained for different caloric and non- caloric beverages. The consumption of milk declined with age. Boys drank more milk than did girls. The consumption of soda, sports and energy drinks, and tea and coffee increased sharply with age, but was not affected by gender.

Only the consumption of 100% juice increased with income. No effect of IUC was observed for any other beverage shown. While lower income groups consumed more tea and coffee, the effect was not statistically significant. Whites consumed much more flavored water and fruit drinks than did non-whites. Milk consumption was higher in Scotland, Wales and Northern Ireland than in the South of England. Overweight and obese children drank more water and beverages than normal-weight children; however trends for fruit drinks and soda were not significant at 0.01 level.

### Water and beverage consumption by time of day and location

**Fig. 1:**
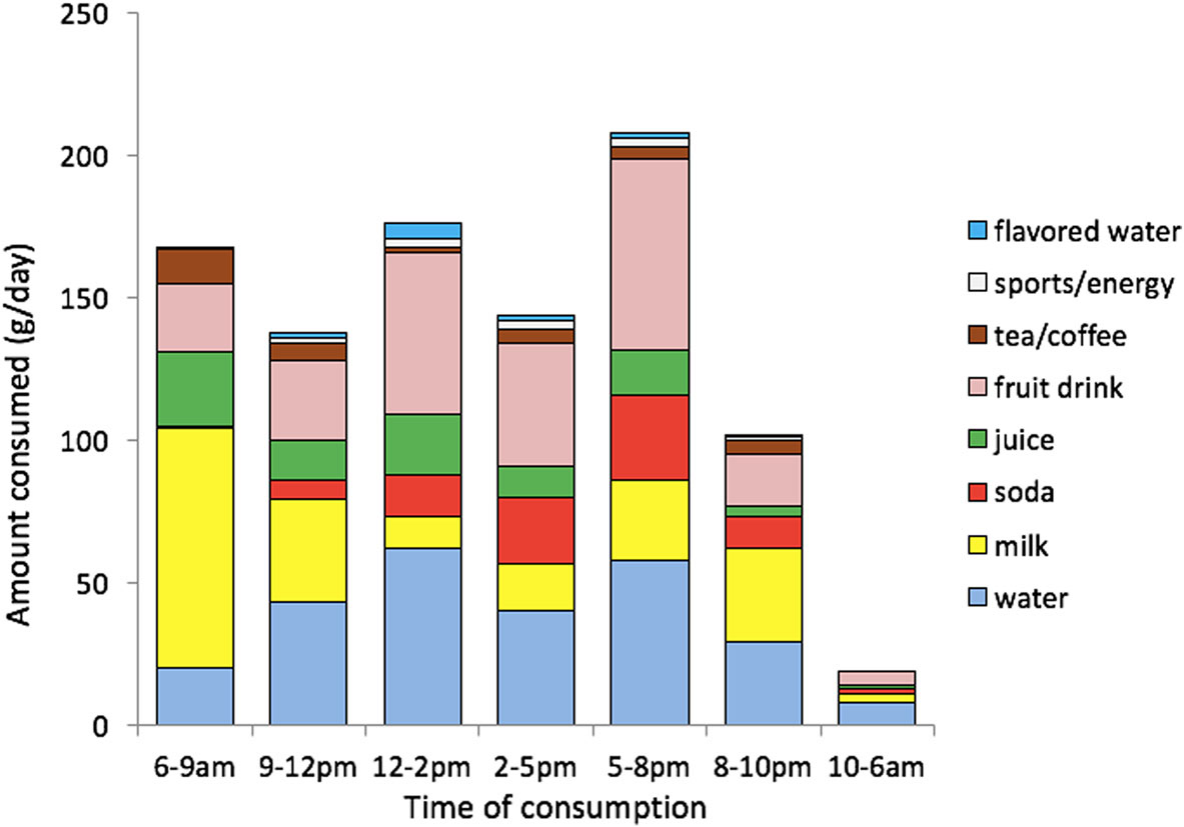
Time distribution of water and beverage consumption by beverage category for children aged 4–13 years

Figure 1 shows that most water and beverages was consumed between 5 pm and 8 pm (207 g/d), corresponding to dinner time, followed by times corresponding to lunch (176 g/d), and breakfast (167 g/d). Beverage choices shifted by time of day. Plain water consumption was higher between 12 pm to 2 pm (lunch) and between 5 pm to 8 pm (dinner) than at any other time. Breakfast was the occasion to consume milk (84 g) and 100% juice, followed by fruit drinks and water. Tea and coffee were mostly consumed from 6 to 9 am (12 g). Water (62 g), fruit drinks (57 g) and juices (21 g) were consumed at lunch. Fruit drinks (66 g), water (57 g), soda (30 g) and milk (28 g) were consumed around dinner time (5–8 pm). Milk came back between 8 and 10 pm (33 g). Most sugar sweetened soda was consumed at dinner time (30 g) and during the afternoon hours (23 g). Beverage consumption at night (10 pm to 6 am) was very low.

Breakfast was thus characterized by the consumption of milk, 100% juices, and tea and coffee. Lunch was the occasion to consume fruit drinks, water and 100% juice. Morning snacks favored milk over fruit drinks, whereas afternoon snacks (between 2 pm to 5 pm) had less milk but more fruit drinks and soda.

**Fig. 2:**
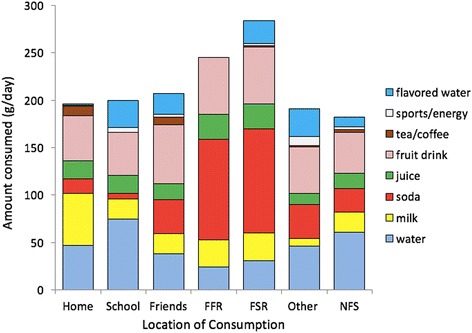
Water and beverage consumption by location. The most common reported location was home (*n* = 845), followed by school (*n* = 640), friends (*n* = 360), FFR (*n* = 110), café restaurant (*n* = 219) and other (*n* = 583). Abbreviations: FFR- fast food restaurant; FSR- full service restaurant; NFS- not further specified

Figure 2 shows water and beverage consumption by location. Each NDNS respondent could list multiple locations for beverage consumption; per consumer analyses were based on declared respondents for that location only. The most common reported location was home (*n* = 845), followed by school (*n* = 640), friends (*n* = 360), Fast food restaurant (FFR, *n* = 110), café restaurant (*n* = 219) and other (*n* = 583).

For example, all 845 children consumed a beverage at home over the 4 days of NDNS. Average consumption was 47 g of water, most of it from the tap (43 g). More water was consumed at school (75 g), most of it also from the tap (60 g). Less tap water was consumed in a fast-food (14 g) or in a café (21 g). By contrast, less bottled water was consumed at home (5 g) than at school (15 g) or other places (20 g).

Whereas total beverages consumption was highest in café-restaurants (261 g) and in fast-foods (244 g), milk was mainly consumed at home (55 g). By contrast, sodas were consumed away from home, mostly in café-restaurants (110 g) and in fast-foods (106 g). About 40% of beverages consumed in restaurants were sodas. Tea and coffee were more likely to be consumed at home and at friends’ homes, whereas 100% juices were more likely to be consumed in restaurants (see Fig. 2). Whereas the consumption of fruit drinks was ubiquitous, more flavored water was consumed away from home. The consumption of sport and energy drinks was very low.

### Total water intake from drinking water, beverages, and food moisture

Table 3 shows the main sources of total daily water intake (ml) by age, gender, ethnicity, season, BMI and socio-demographic group. Mean total water intake from plain water, beverages and foods was 1338 ml. Water and beverages together contributed 901 ml (67%), with beverages accounting for 48% of it, whereas food moisture contributed 437 ml (33%).

**Table 3:** The consumption of water from water and beverages and from food moisture by children in the NDNS sample (*n* = 845) by age group, gender, and socio-demographic group. The data are means and standard deviations (Std)

	Water Total			Water + Beverage		Food moisture	
	N	Mean	Std^1^	Mean	Std^1^	Mean	Std^1^
All	845	1338.2	400.6	901.2	355.8	437.0	133.0
Age (years)							
4-8	436	1278.5	350.8	845.9	304.2	432.6	126.5
9-13	409	1396.3	439.7	955.0	396.1	441.3	139.5
		**<0.0001**		**<0.0001**		0.4122	
Gender							
Boys	430	1359.4	390.2	913.6	347.4	445.7	135.2
Girls	415	1316.0	410.3	888.1	364.3	427.9	130.1
		0.1548		0.3352		0.0618	
Quartiles of IUC							
Q1 (poorer)	148	1216.0	366.0	812.0	321.0	404.0	137.5
Q2	186	1372.5	431.3	940.7	390.5	431.7	133.4
Q3	215	1358.5	380.6	913.5	327.8	445.0	133.5
Q4 (richer)	197	1431.7	417.4	966.5	382.3	465.2	122.9
		**<0.0001** ^**abc**^		**0.0002** ^**ac**^		**0.0015** ^**c**^	
Season							
Winter	230	1305.8	359.8	868.8	303.2	436.9	122.7
Spring	233	1376.8	409.9	944.2	371.7	432.5	131.3
Summer	207	1368.0	407.6	926.3	364.7	441.6	138.5
Fall	175	1289.7	424.5	851.8	379.5	438.0	142.2
		0.0836		0.0337		0.9305	
BMI							
Under/normal	607	1330.6	409.5	891.3	359.5	439.3	135.1
Over/obese	204	1356.7	368.9	929.4	343.8	427.3	127.8
		0.4514		0.2110		0.2944	
Ethnic group							
White	723	1358.1	391.5	918.6	347.8	439.4	132.9
Non white	122	1237.2	436.4	812.6	387.7	424.6	133.2
		**0.0063**		**0.0088**		0.2559	
Region							
England: North	201	1313.2	365.2	880.4	316.2	432.9	129.3
England: Central/Midlands	142	1360.4	418.6	915.8	365.0	444.7	122.7
England: South (incl. London)	355	1323.0	411.0	889.6	370.7	433.3	132.2
Scotland,Wales, Northern Ireland	147	1395.8	401.2	949.8	360.2	446.0	149.0
		0.2906		0.3254		0.7449	

^a^Significant two by two differences: Q2 vs Q1

^b^Significant two by two differences: Q3 vs Q1

^c^Significant two by two differences: Q4 vs Q1

^1^Std^:^ Standard Deviation

Comparisons between two means were either significant (bold) or not

Older children had higher total water intake (1396 ml) than did younger children (1278 ml). There was no difference between boys and girls. There was a sharp effect of incomes: highest income groups had higher total water intake than did lowest income groups. White children consumed more total water than did non-white children. There were no differences in total water intake by BMI, seasonality, or region.

The effect of age, but not gender, was observed for combined water and beverages. Higher consumption was observed for Whites and the highest income group as compared to the lowest.

**Fig. 3:**
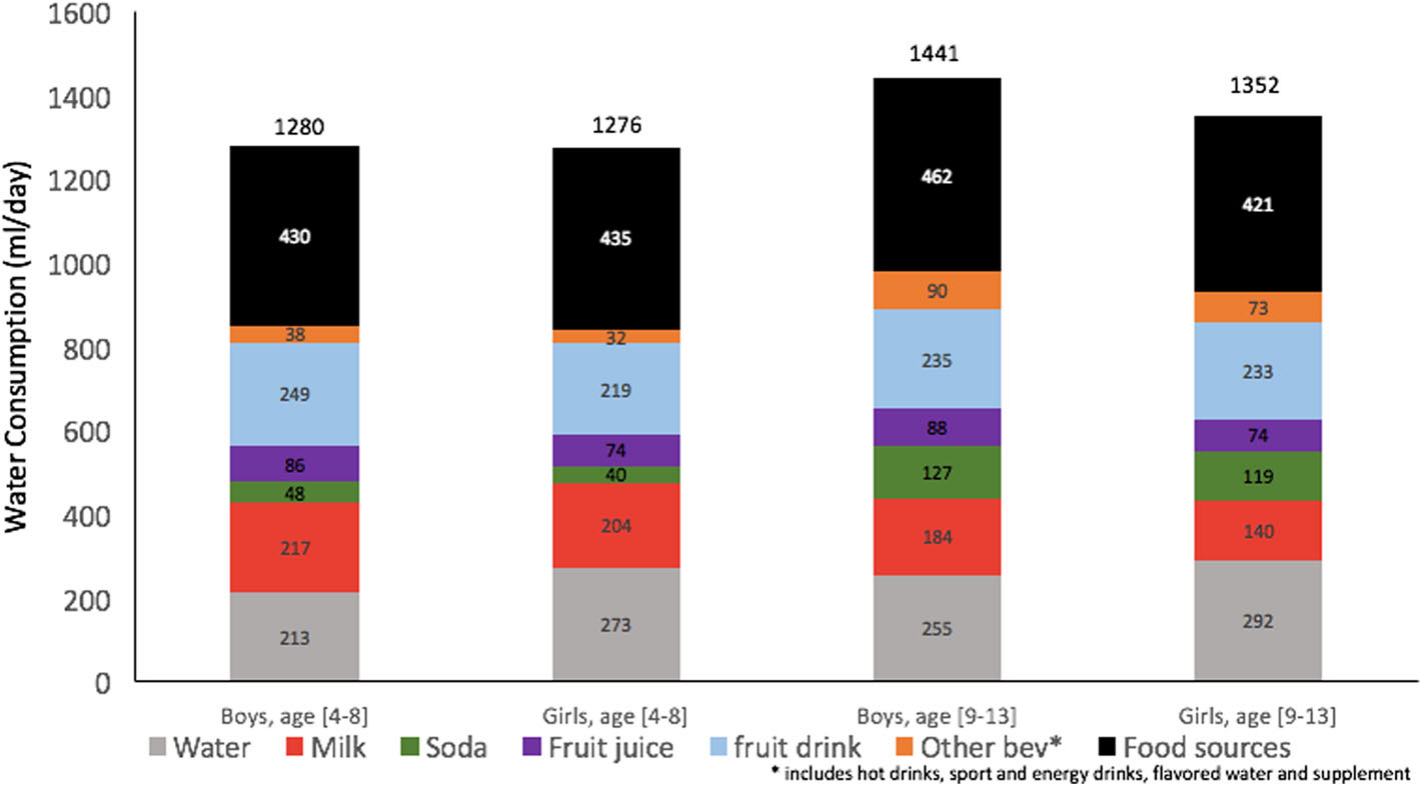
Mean total water intake (ml/d) from different sources (water, beverages and foods) by age and gender

Figure 3 shows total water consumption according to the age by gender categories. As previously reported, milk consumption for 9–13 year old girls was significantly lower than for 4–8 year old children (boys or girls). Conversely, older boys and girls (9–13 years age group) drank significantly more soda than did younger boys and girls (4–8 years age group).

### Meeting EFSA recommendations

**Fig. 4:**
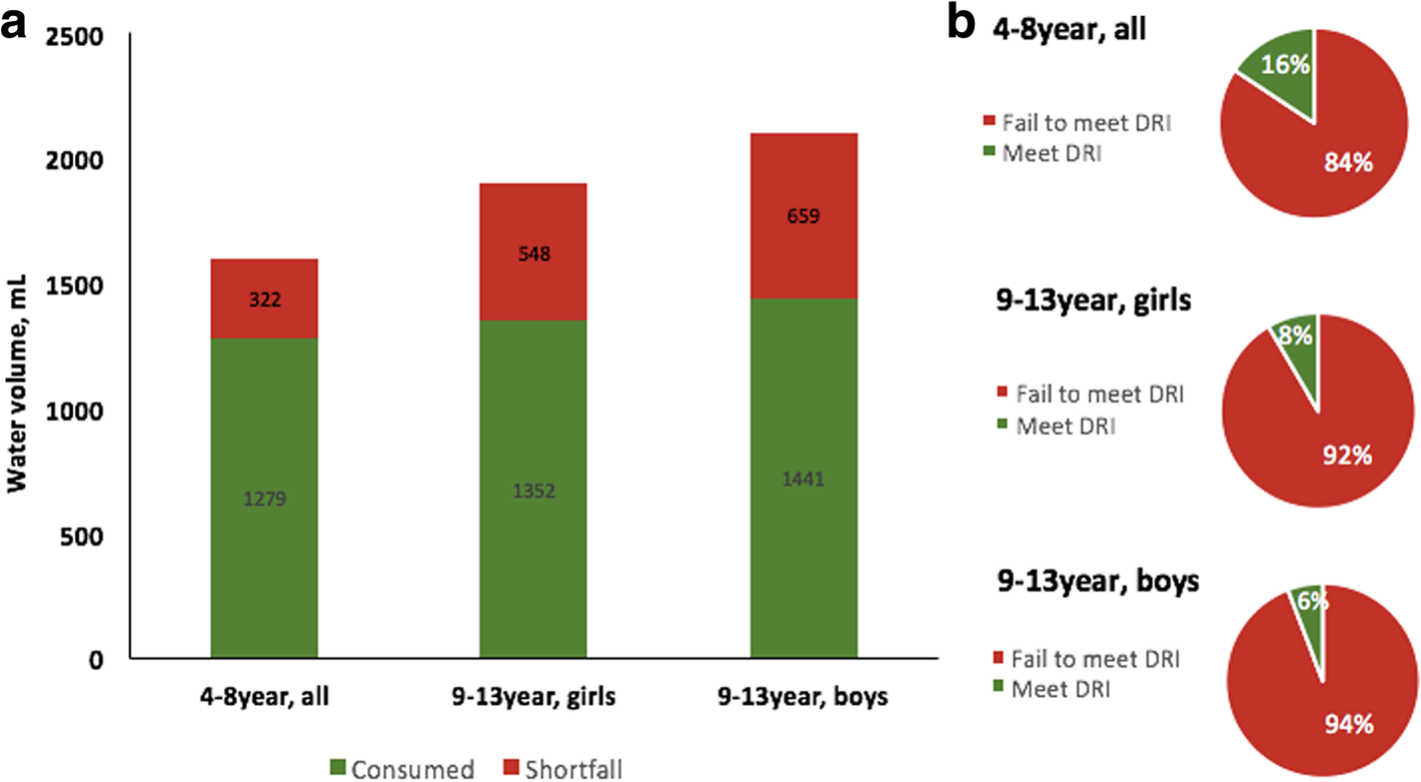
Total water intake and deviation from the EFSA recommendation and percentage of children who met the EFSA recommendations by age and gender. **a** Total water intake and shortfalls (in ml) relative to EFSA recommendation by age and gender. **b** Percentage of children falling to meet EFSA recommendations by age and gender

Total water intake in relation to the EFSA standards are shown in Fig. 4. The shortfall in water consumption is indicated as well. For, 4–8 years old boys and girls, the shortfall was 322 ml, whereas for 9–13 years olds it was 548 ml for girls and 659 ml for boys. The percent of children who failed to meet EFSA guidelines was 88.7% (84.4% for 4–8 years group and 92.8% for 9–13 years group). Mean total water intake among children aged 4–13 years covered only 74.8% of EFSA recommended amounts (79.9% for younger children and 69.9% for older children).

Additional analyses calculated total water intake per 1000 kcal by gender and age. Overall, the ratio was 0.845 L/1000 kcal (0.83 for boys and 0.86 for girls). No significant differences by gender were observed after adjusting for energy.

## Discussion

The present study adds to the existing literature on water consumption in the UK [8] and allows for a direct comparison of children’s water drinking habits in France and the US [5, 27]. Those prior analyses were also conducted using nationally representative databases collected by the respective governments. Dietary intake methodologies were comparable. Whereas the French INCA data were based on 7-day food records, the British NDNS used 4-day food records. Our past studies, conducted in the US and in France [5, 26], used the same age groupings (4–13 years), the same beverage classification scheme, and also distinguished between tap and bottled water. Total water intakes were compared to adequate water intake by age group, as defined by EFSA and by the US Institute of Medicine [5, 26].

Plain water consumption in UK was estimated at 258 g/d. The bulk of water came from the tap (84%); only 16% came from bottled water. By contrast, plain water consumption in France was estimated at 453 ml/d, of this 242 ml came from the tap (54%) and 210 ml (46%) from bottled water [26]. In the US, plain water consumption among 4–13 year olds was 431 ml/d, of this 257 ml (59.6%) came from tap and 174 ml (40.4%) from bottled water.

Plain water consumption among UK children varied by household income. The effect of income was observed for total and for tap but not for bottled water, in part because of low levels of consumption of bottled water. A similar income gradient for tap water consumption was observed in the US; it was significant for adults but only marginal for children [4, 5]. By contrast, water consumption (tap and bottled) among French children was much higher overall and no income gradient was observed [26].

Beverage consumption patterns in the UK also depended on incomes. Higher income groups in the UK consumed more water and more beverages than did lower-income groups; the effect was driven by tap water and by 100% fruit juice. Lower income children tended to consume more tea.

Total water consumption from plain water and beverages was 901 g in the UK, 801 g in France, and 1136 g in the US [5, 26]. Patterns of beverage consumption varied by age. Milk consumption declined with age whereas the consumption of sweetened beverages increased, consistent with other data from France [26, 27], the UK [16], and the US [5]. Total water intakes in the UK, but not in France, varied with income.

The consumption of sweetened fruit drinks by UK children (242 ml/d water) was significantly above US values for regular fruit drinks (79 ml/d water) and was more than double the consumption of soda (89 ml/d water). By contrast, French children derived water from drinking water, then milk, then 100% juice and only then soda [26]. The consumption of sweetened fruit-based drinks in France was negligible [26], in marked contrast to the UK. In the US the children drank more soda than either fruit juices or fruit drinks [5].

There were only minor differences by race/ethnicity in the UK . By contrast, the patterns of tap versus bottled water consumption in the US varied by age, gender and race/ethnicity [5, 7]. Non-Hispanic white children drank more water than did Mexican American and non-Hispanic black children.

Analyses by time and place of consumption allowed for further insights into UK children’s water drinking habits. In the UK, water and sweetened beverages were mostly consumed at lunch and dinner times; the dominant beverage at breakfast was milk. Fruit drinks equaled or exceeded plain water consumption between 12 noon and 10 pm. By contrast, French children drank virtually no fruit drinks at all [26]. Breakfast was characterized by milk and 100% fruit juice almost exclusively [26]. The dominant beverage at lunch and dinner in France was plain water, bottled and tap, whereas soda consumption was low [26]. Clearly, there are major cultural differences between the UK and France with respect to what beverages are viewed as appropriate for children’s meals and snacks. A similar analysis by time and place of consumption for the US would be of great interest.

British children drank most milk at home and most plain water at school. By contrast, beverages consumed at full service and quick service restaurants tended to be soda and fruit drinks and to a much smaller extent plain water and milk. The present data would suggest that British children appeared to consume more beverages away from home than did French children. Bellisle et al. [13] had previously noted that French children consumed about 80% of the total daily fluid intake at home [13]. However, data on the place of consumption ought to be interpreted cautiously, given many instances of places identified as other or unspecified.

Total water consumption from water, beverages, and food moisture in the UK was estimated at 1338 ml/d. Of this 901 ml was provided by water and beverages and 437 ml/d by moisture from foods. Total water consumption in France for the same age group was estimated at 1324 ml/d – a wholly remarkable level of agreement. Of this, 801 ml was provided by beverages and 524 ml by moisture from food. Total water consumption in the US was estimated at 1580 ml. Of this, 1136 ml was provided by water and beverages and 447 ml by food moisture.

French children derived more water from food moisture (39%) than did children in the UK (33%) or in the US (28%). That may say something about the quality of the diet, given that, in general, higher levels of moisture from food point to a lower energy density diet with more vegetables and fruit.

Comparisons of water intake from all sources to EFSA guidelines were consistent with past findings. Water intake in UK covered only 74.8% of EFSA recommendations and 88.7% of all children failed to meet the guidelines. Very similar figures had been obtained for France: guidelines were not met by 89% of all 4–8 year olds and 90% or more for 9–13 year olds. In Belgium non-compliance was 93% for girls and 92% for boys in the 9–13 years age range [12]. By contrast, fewer US children failed to meet the (higher) recommendations issued by the US Institute of Medicine. Indeed, 85% of the recommendations were covered for 4–8 years old children, 78% for 9–13 years old girls and 73% for 9–13 years old boys. Failing to meet the guidelines were 75% of 4–8 year olds and about 84% of 9–13 year olds [5]. Of course, it needs to be stressed that not meeting guidelines does not imply dehydration or any adverse medical condition; merely that the existing guidelines for adequate water intake are not being met.

Replacing caloric beverages with plain drinking water is a public health priority in the UK, the US and in France [1, 2]. UK based studies [17], using a longitudinal cohort, have already examined the impact of sugar-sweetened beverage (SSB) consumed by 5–7 year olds on BMI values at 9 years of age.

The cultural differences in water drinking habits between the UK and France may suggest new avenues for intervention. First, French children consumed much more plain water, tap and bottled [26], than did UK children whose consumption patterns ran toward sweetened beverages, mostly fruit drinks. Second, plain water was the almost exclusive beverage of choice at lunch and dinner in France [26]; not the case in the UK. Third, water, both bottled and tap, was the almost exclusive beverage in French schools, again, not the case in the UK. As noted before, the consumption of plain tap water in the UK was higher among higher income households; no comparable social gradient was observed in France [26]. By contrast, consumption habits by time and place were comparable. Beverages were consumed mostly with meals. Whereas milk was consumed at home, sweetened beverages (sodas, fruit drinks) were consumed outside, principally for afternoon snacks and lunch.

The study had limitations. First, the NDNS data, based on self-report, are subject to random inaccuracies and to systematic reporting biases. The proxy recall for younger children may be an additional source of bias. The 4 days of food records impose a burden on respondents and data quality can be variable. The NDNS database is the standard source of information about dietary intake in the UK and is comparable in scope and importance to the National Health and Nutrition Examination Survey in the US and the INCA 2 database in France.

## Conclusions

The present analyses represent one of the first explorations of the consumption of drinking water relative to sugary beverages by children in the UK and suggest potential avenues for intervention. Based on cumulative evidence from multiple countries [8, 15], it seems reasonable to promote the consumption of plain water as the beverage of choice to meet the daily hydration needs. Healthy food and beverage patterns, developed during childhood, are an integral component of a healthy lifestyle.

## Abbreviations

BMI: Body Mass Index
DRI: Dietary Reference Intakes
DRV: Dietary Reference Values
EFSA: European Food Safety Authority
FFR: Fast Food Resturant
FSA: Food Standards Agency
FSR: Full service restaurant
HNR: Human Nutrition Research
INCA: Étude Individuelle Nationale des Consommations Alimentaires
IUC: Income per Consumption Unit
NatCen: National Centre for Social Research
NDNS: National Diet and Nutrition Survey
NFS: Not further specified
OECD: Organisation for Economic Co-operation and Development
PHE: Public Health England
Std: Standard Deviation
UCL: University College London
